# Broke in the War; How the Wounded Japanese Are Cared for

**Published:** 1905-12

**Authors:** J. Gordon Smith

**Affiliations:** Correspondent for the London Morning Post with General Oku’s Army


### Broke in the War; How the Wounded Japanese are Cared for.

By J. GORDON SMITH.

Correspondent for the London Morning Post with General Oku's Army.

[*Canada Lancet.*]

ON a dull-grey morning I stood before Shinbashi station and watched the wounded arriving in Tokio; also gun-carriages, limbers, horses, and other trophies of war. There was more interest displayed by the throng which jostled me about the trophies than the wounded. The govermental practice is to send each batch of broken men to the divisional headquarters. These, whom I saw, were two officerrs and three hundred and forty-one men of the Imperial Guards of Tokio. About three months ago these men were entrained at night and went away without flare of trumpet, without any demonstration, steaming away under cover of darkness to land at night by the light of hundreds of torches on Korea's shores. Now they have returned with equal lack of demonstration.

Their home-coming was a sad sight. Under a leaden sky, on a dull, grey, depressing day they came from the train quietly and with no more show than if they had been a party of farm laborers returning from the rice fields south of the city. A great crowd was at the station but there was no welcoming shout. All was done in the most dispassionate business-like way. The majority walked more or less briskly from the platform carrying their goods packed in blankets and in haversacks slung from their shoulders; the greater number were smoking cigarettes. Some were limping, their hands on the shoulders of others. A comparative few were carried on stretchers. Hundreds of 'kuru-maya' with their little two-wheeled 'jinrikishkas' (literally translated,'man-power cars') were standing in lines before the porch of the railway terminus, and, one by one, they were called by an officer of the medical corps to have a returned soldier seated in their little passenger carts. From the goods platform some Russian gun-carriages, three big horses—seeming large in comparison with the small Japanese ponies—some gun-limbers and ammunition wagons, and other trophies of the battle of the Yalu, were dragged out. The line of 'jinrikishkas' fell in behind.

It was a procession of strange contrasts. The silent people by the roadsides saw the spoils of war they prized so much—the Japanese are very fond of the display of trophies such as these—and they were pleased. The gun-carriages passed; the limbers rolled by. Behind were coolies with mushroomlike hats and blue blouses, jogging on with the wounded. The red crosses on their white canvas kimonas and hats, such as those of pastry-cooks, were not needed to manifest the other side of war—the seamy side. One after another the broken soldiers were trundled past the onlookers, many bare-headed, many with white hospital cap worn over their yellow braided regimental cap, the hospital kimona over their foreign cut uniforms; all with crimson blankets slung from their left shoulders, in striking contrast with the white garments. Some had their heads bandaged, others had arms or legs bound in lint. The brown faces all had a pallor; they were a pitiable sight.

The procession was a long one, stretched out over a mile. Hundreds stood on the streets, not crowding in any place other than at the station, but in an almost continual line on each side of the roadways, each person absolutely silent. The soldiers themselves seemed to take little interest in their surroundings, looking at the landmarks about them with indifference. The sentinels at the gates of the Russian legation; the officials at the windows of the War and Naval Departmental buildings,—all else they saw on the way, had, seemingly, no interest to them. The old fellow with great smoked glasses giving him the appearance of a sage, who paid the 'kurumayas' at the hill-top, giving each coolie a ten sen piece and two sens—which allotment by the War Department for the transport of the sick and wounded were arranged in little piles on a big tray; the War Minister who drove past, his uniform glittering, in his carriage from the General Staff office, the 'gogai-runners' rushing by the little carts clanging their bells and shouting the name of the newspaper whose extras they sold; none of these things had, as far as one could see, any interest to the wounded man. They were impassive.

The Eyu Hospital of the medical corps stands on the brow of a hill, not a stone's throw from the General Staff office, where the generals were sitting at a board of strategy, devising new battles that would make more wounded even as the 'jinrikishkas' were rolled into the yard. Across the roadway from the ponderous gate which swings from two great beams joined by an equally massive beam overhead, is the moat, beyond is the old stone wall of the feudal days, with its overhanging trees hiding the palace buildings. The Tenshi Sama, for whom the men who had been injured in battle had gone to fight, and were eager to fight again, lived beyond that wall.

The hospital is a one-storied structure, square as a box other than for the wide porch, the curved roof of which, with a sweep, tiling, carvings and scrolled panels as pretty as those of a temple, gives the building a picturesque effect. Without this porch with its central panel of a sixteen-leafed chrysanthemum—the crest of the Tenshi Sama—the building would be a barren looking barn; with it the place is picturesque and pleasing. On the afternoon of that grey day when the several unfortunates broke in the war were squatted on the stones and grassy banks of the drive-ways as a spectacled doctor read the roll, the picturesqueness of the place was increased. The wounded squatting about added much to the effect.

All were kept sitting before the hospital entrance for nearly two hours. Everything was done with a system that was remarkably complete, often too complete, for regulations are sometimes carried to an absurd limit. The soldiers had all been landed at the hospital entrance and the doctor was calling the roll while assistants booked the names, when carts came with further supplies of hospital clothes. "Rikishkas" arrived with more doctors, and then came many carts, drawn by horses and oxen, bringing heaped loads of wooden bunks, the peculiar box-like sleeping places of the Japanese soldiers in garrison. Three hundred and forty-one beds were moved into the almost bare rooms of the hospital by nightfall, and meanwhile the sick and wounded sat outside conversing with each other and smoking cigarettes, exhibiting bullets from Russian rifles and telling and retelling the story of the battles for the benefit of the men of the medical staff—and telling also of the disappointment each man felt at not being allowed to remain at the front. Some spoke emphatically of wrong done them in ordering their return. They were aggrieved for they felt that they were still fit to fight. Those who had practically recovered during the voyage home in the hospital ship had petitioned to be permitted to return even when en route home; all longed to be back.

I will long remember the scene, it was so very impressive. There was so much to be seen in the faces of the men who sat there, expressions of indifference, of fatigue, of hope, of sorrow—all the emotions were there displayed, but held well in check, for a Japanese will ever mask sorrow with gladness if others watch. This is the way with all. I saw a mother and brother greet a wounded soldier amidst the throng who sat before the hospital. His hand was bandaged, his arm swollen, and his face was as pallid as a brown complexion can show pallor. Yet he smiled, his white teeth showing. There were no tears in his eyes, and no outburst of joy, no emotional display of any kind marked the coming of those he loved. The old woman, with a well-worn grey kimona bound close about her, shuffled over the pebbles with her high "geta," and her other son, the carpenter—the tradesman has a mark of his guild shown by the great ideographs monogrammed on the back of his coat—walked behind her. Neither displayed any feelings; the other soldiers were sitting by and it is not in public that the emotions are to be displayed. The soldier must not be shamed before his fellows. The calm exterior must mask the feelings, no matter what one does behind the paper-screened walls of the home. So the soldier bent his back, bowing ceremoniously, and the mother and brother bowed equally low and with equal form. They spoke in polite commonplace words as they greeted each other and, bowing again, separated. Imagine, if possible, an Anglo-Saxon mother receiving a wounded son without even a hand clasp. Yet that is the Japanese custom. Many were received by friends and relations, as I watched, with lack of emotional display. It is this seemingly restrained manner which the Japanese adopt in public, when whatever one feels, the indications of the feelings must be suppressed, that has given the foreign observer the impression that the Japanese are undemonstrative in their affections; that they lack emotion. But this is not so. In public the Japanese is undemonstrative. One would never think of showing any affection in public; that is for the home. And so, all who come to visit the wounded—people came and went until dusk—were received with ceremony. Meanwhile, the doctor who stood behind the table, placed on the steps at the entrance and heaped high with books and cards—there was a card for each man—called the soldiers one by one, and, with a parting bow and hurriedly spoken "Sayonara," each man hurried into the building. There, the pharmaceutists were busy distributing the medicines the doctors prescribed. It was nightfall before all were housed, and, as one of the dispensers informed me, it was morning before the work of attending to the reception of the men was completed. The doctors did not even have time to prepare afternoon tea for the volunteer nurses who had come from England and America; they did not have time to attend to the social requirements these ladies sought for many days. In time, though, they were able to send many of these men who came to them from the front, back again to fight for the Emperor—and for Japan.

I visited the hospitals of Tokio where the "men broke in the wars" were being treated, before I left for the front to join General Oku's army, and my experiences impressed me with the fact that the Jepanese army surgeons are demonstrating to the satisfaction of medical men sent to Japan by various nations to study their methods of dealing with the sick and wounded, that more men recover from wounds when operations are not performed than otherwise. With the armies of Japan now in the field the surgeons are operating in very few cases; in no case do they operate until the second day, and then only in cases of extreme urgency. In the main, the wounds of those shot in the field are dressed antiseptically by the surgeons at the front and the dressings are not removed until such time as the soldiers are brought to a hospital where the conditions are perfect for the treatment of the wounded. Even then, there are few operations. The wounds are bathed with an antiseptic washing, and then, as an American army surgeon whom I met at Sekijuji, or Red Cross Hospital, said "they let the Lord do the rest—and He does it."

At both hospitals, the Biju Byoin, or military hospital, and the Sekijuji, or Red Cross Hospital, I saw how successful indeed was the Japanese method of treating the wounded. The high percentage of recoveries in comparison with the records of other armies in past campaigns is convincing that this policy of "laissez fairs" adopted by the Japanese military doctors is accomplishing wonderful results. Both hospitals are single storied buildings with long narrow wards, windows and rows of beds on either side; the ventilation is excellently arranged and everything is spotlessly clean and sweet smelling. There are no bad odours. The percentage of recoveries was remarkable. I saw a large number of wounded who had perforating wounds in the chest going through the pleural cavity, yet not a case of pleurisy resulted. I also came in contact with some six cases of perforating wounds that passed through the abdominal cavity and out of the back, and, although the wounds were received not more than five or six weeks before, some of the men were sitting up in bed; two were walking about convalescent and complaining of the delay in permitting them to return to the front. True, the worst cases were probably not seen in the hospitals of Tokio. The men sent there, I understand, are selected from the cases brought to the southern depots by the hospital ships. But, nevertheless, the results secured by the surgeons are remarkable. The wounds I saw were nearly all clear perforations, and, unlike some bullet wounds I have seen, the orifice of exit was no larger nor less clear than the orifice of entrance. There was no suppuration. I saw a bullet taken from a man's jaw and the jacket was perfect. The bullet had evidently been spent when it struck the soldier and had been stopped on striking the lower bone of the jaw. It differed little in size from the bullet used by the Japanese and was a smooth, pointed, compound metal-jacketed ball. The doctor who accompanied me offered the bullet to me, but the soldier was emphatic in his suggestions; he wanted the bullet as a souvenir and I gave it to him.

There were some remarkable cases. One soldier with whom I spoke, aided by an interpreter, had been struck by a bullet just under the left eye, where the orifice was plainly visible, and the bullet had passed through the sphenoid bone and perforated the tissue, coming out below the scapula of his right shoulder. His only suffering was from slight paralysis of his right arm, due to the fact that the bullet had broken one of the nerve tissues. And, although not more than forty-five days had elapsed, the soldier was able to tell of how he had been shot when charging with his comrade on the Russian position at Hohmatung. A more remarkable case seen at the military hospital was that of a man who had received a bullet in the forehead which had come out at the back of his head, both orifices being plainly shown, and he not only lived, but was sitting up in bed able to tell of his wound. He gives the credit of his recovery to a talisman in the shape of a samisen string which a geisha had tied about his waist. Another soldier received a bullet under his chin and left the top of his head; yet he was recovering. If the orifices made by the bullet were not so plain it would have been difficult to believe that possible. It seemingly is. The little spectacled doctor pointed out many instances in his text books; some of which are printed in English, some in German.

I met Surgeon-Major L. L. Seaman, of the U. S. Volunteers, at the Sekijuji-sha Byoin, and as we left to get into our 'jinrikishkas' he said to me, "After what I have seen, I would hesitate to operate on a single case at the front." The feature of the Japanese surgeons' work is that he leaves the wound alone; there are few operations, indeed, almost none at all. Of course there are some cases and such things where the knife is used, but it is used no more than is absolutely necessary. The "first aiding" dressing of the Japanese is very simple, and when it is placed on the wound by the surgeon at the front it is not touched again until a hospital is reached. The wounds are usually aseptic. Sometimes the wounds are jagged, the detachment of the jacket or introduction of foreign matter, cloth, button, etc., or the inpingment or ricochet of the bullet being responsible for such wounds. These are in the minority, though, for the greater number of wounds I saw in Japanese soldiers—and I saw hundreds in the field—had very minute orifices, those of entrance and exit being hardly distinguishable from each other in appearance. The Japanese believe it is far better to bandage a wound properly and avoid infection than risk danger by an operation under such conditions as prevail in the field. The Japanese are ever apt pupils and they are following well the examples set by Lister and Pasteur, to whom military surgery owes its greatest debt, and the Mikado's surgeons hold that the soldier who falls on the battlefield from the effect of a ball passing through any vital part of his anatomy and who has a "first aid" bandage promptly applied and is then transported to a general hospital where the Röntgen ray and the principles of asepsis and antisepsis can be utilized, has a far greater chance of recovery than when his wounds are treated on the field. In the war between the United States and Spain, the United States forces had 95.1 per cent. recoveries, while 4.9 per cent. died as a result of following these conservative methods. The Japanese have even better results.

While with General Oku in Manchuria I saw much of the work of the Japanese surgeons. They have much to do in keeping the armies under their charge up to the highest standard of health, so that in the emergency of battle the soldiers may be fitted to do their duty. The surgeons are also sanitary engineers, and they select the sites for camps, arrange camp drainage, locate latrines, and inspect all water supplies. It is the rule of the Japanese armies in the field to send a corps of medical experts in advance of the army, and, before the army pitches camp, every water supply in the vicinity, every well, has been chemically analyzed. Placards are placed at all places where there is water. Some of the placards read "This water is good," others, "This water is bad" and still others, "This water should not be used unless it is boiled for half an hour." These precautions and the good ration in use prevents intestinal troubles and there are few cases of intestinal affections.

While I am on the subject of hospitals, a few words regarding the Japanese Red Cross Society might not be without interest. The Red Cross Society in Japan is an outcome of the Hakuaisha (Society of Benevolence) founded during the Satsums rebellion, the great civil war of 1877. At the close of the rebellion, the society was constituted a permanent organization, and, when Japan recognized the Geneva Convention, the Society of Benevolence procured a connection with the international committee at Geneva and was merged into the Red Cross Society of Japan. Now the society has over 800,000 members. An Imperial Prince is the honorary president and a Princess of the Blood, head of the ladies' committee. H. I. M. the Empress is a constant visitor and patron of the hospitals of the society. Barons Ishiguro and Hashimoto, prominent Japanese medical men, are among the moving spirits of the society. The headquarters is in the capital, and consists of a number of buildings for central offices and storerooms. One room, elegantly furnished, is set apart for the use of the Empress or Emperor, and in this room are excellent full length oil paintings of their majesties. The society's hospital, the Sekijuji-sha Byoin, situated in Shibuya suburb, has 230 beds ordinarily, but more have been put in to accomodate the wounded that have arrived there. The Empress, who is a frequent visitor, has a room set apart for her at the hospital. The nurses, all gowned in white with an odd, high-crowned cap, number two hundred and sixty in all. The storerooms are large warehouse buildings, each laden with an enormous reserve supply of hospital stores, stacks of lanterns, canteens, uniforms, blankets litters, trains of ambulance, cots, dressing material, etc. The society is the richest in the world. Within two days it can load a hospital ship or a hospital train, in readiness for the front. There is not the slightest confusion, the system being excellent. The nurses are all under military control. Two hospital ships are owned by the society, the Kakuai Maru and Kosai Maru. In peace time they are leased to the Nippon Yusen Kaisha as passenger vessels. Now they are engaged in carrying the sick and wounded back to Japan from the front.

